# *Bacillus pumilus* AD14: A Saline-Alkali-Tolerant Plant Growth-Promoting Bacterium for Enhancing Soybean Tolerance and Ameliorating Saline-Alkali Soil

**DOI:** 10.3390/microorganisms14061168

**Published:** 2026-05-22

**Authors:** Changjun Zhou, Yiqing Chen, Ying Yu, Bing Liu, Jidong Yu, Yaokun Wu, Jianying Li, Lan Ma, Gang Chen, Xu Feng

**Affiliations:** 1 Daqing Branch, Heilongjiang Academy of Agricultural Sciences, Daqing 163311, China; andazhouchangjun@163.com (C.Z.); liubing528@163.com (B.L.); yujidong666@126.com (J.Y.); wuyaokun530@126.com (Y.W.); lijiangyingfz@163.com (J.L.); malan042999@163.com (L.M.); 2 College of Life Sciences, Northeast Agricultural University, Harbin 150030, China; chenyiqing0327@163.com (Y.C.); 18045043687@163.com (Y.Y.); 3 Daqing Agricultural and Rural Social Undertakings Service Center, Daqing 163311, China; dqzzwfz@163.com

**Keywords:** saline-alkali soil, PGPB, soybean, *Bacillus pumilus*, growth-promoting ability

## Abstract

According to an FAO report, the total area of saline-alkali land worldwide is approximately 954 million hectares, accounting for about 20% of global cultivated land. Saline-alkali stress significantly reduces soybean (*Glycine max* L.) yield and quality, and saline-alkali-tolerant plant growth-promoting bacteria (PGPB) have shown important application value for soybean planting in such farmlands. In this study, 15 strains of saline-alkali-tolerant bacteria were isolated from saline-alkali soil in Anda City, Heilongjiang Province, China, and identified morphologically, belonging to the genera *Enterobacter*, *Bacillus*, *Chryseobacterium*, *Acinetobacter*, *Enterococcus*, and *Pseudomonas*. Through tests for nitrogen fixation, phosphorus solubilization, potassium solubilization, hydrolase production (including pectinase, amylase, and protease), and germination promotion assays, *Bacillus pumilus* AD14 was identified as having the best growth-promoting effect on soybean seedlings. Pot experiments in saline-alkali soil showed that AD14 significantly promoted soybean seedling growth, increasing plant height by 5.63–6.37 cm and root length by 3.58–3.99 cm compared to the control. AD14 also enhanced saline-alkali tolerance by improving the activity of antioxidant enzymes including superoxide dismutase (SOD), peroxidase (POD), and catalase (CAT) and increasing soluble sugar and protein contents. Meanwhile, soil pH decreased by 10.94–12.15% and soluble salt content decreased by 9.59–13.39% after planting, and soil enzyme activities (including urease, sucrase, and catalase) increased markedly. These results demonstrate the great potential of AD14 for soybean planting in saline-alkali soil. This study provides a relevant reference for enriching the resources of saline-alkali-tolerant PGPB and developing new biological agents suitable for soybean planting in saline-alkali soils.

## 1. Introduction

According to a report by the Food and Agriculture Organization of the United Nations (FAO), the total area of saline-alkali land worldwide is approximately 954 million hectares, accounting for about 7% of the global land area. Of this, approximately 20% of arable land is affected by varying degrees of salinization, which severely restricts crop yields and causes huge economic losses in food and agriculture annually [1]. The main causes include natural factors such as high salt content in parent materials, drought, low rainfall, and strong evaporation leading to salt accumulation on the soil surface, as well as human factors such as improper irrigation, poor drainage, and excessive fertilization. Against the backdrop of climate change, the risk of salinization is expected to further intensify [2].

Soybean (*Glycine max* L.) is an important global crop for oil and high-quality plant protein, serving functions as food, feed, and industrial raw material [3]. It is a key crop for ensuring food security and the development of animal husbandry. Soil salinization can inhibit soybean germination, growth, and podding through osmotic stress, ion toxicity, and nutrient imbalance, thereby significantly reducing yield and quality and restricting the sustainable development of the soybean industry [4,5,6]. For crop cultivation in saline-alkali land, technological approaches such as soil improvement, application of chemical conditioners, and selection of salt-tolerant varieties have been developed [7,8,9]. However, in comparison, the biological improvement strategy of using salt-tolerant plant growth-promoting bacteria (PGPB) to regulate the soil microenvironment and enhance plant stress resistance has more green, efficient, and sustainable advantages [10].

Currently, saline-alkali-tolerant and growth-promoting bacteria are generally obtained through a combination of high-salt and high-alkali tolerance screening, growth-promoting function determination, and molecular biological identification. Their application in crops such as soybean has been proven to improve the rhizosphere environment, enhance stress resistance, and significantly boost growth indicators and yield. For example, Miaoxin Shi et al. [11] isolated the salt-tolerant and growth-promoting bacterium *Bacillus cereus* strain FORC047 from lily bulbs. This strain can significantly improve the growth status of lilies in saline-alkali environments and enhance their saline-alkali resistance. The main mechanism is that this bacterium possesses nitrogen-fixing and phosphorus-solubilizing abilities, which provide key nutrients for lily plants. In addition, it enhances plant tolerance to saline-alkali stress by increasing the activity of antioxidant enzymes, thereby alleviating the damage caused by saline-alkali stress. Guoliang Li et al. [12] isolated a salt-tolerant and growth-promoting bacterium, *Peribacillus simplex* M1, from maize roots which can significantly enhance maize seed vigor and effectively improve the growth status of maize seedlings in saline-alkali environments. Its main mechanism of action involves colonizing maize roots through its intrinsic saline-alkali tolerance, enhancing maize resistance to saline-alkali stress by increasing plant antioxidant enzyme activity, and directly supplying essential nutrients for maize growth via its nitrogen-fixing and phosphorus-solubilizing functions. Yamei Gao [13] isolated and screened a strain of *Streptomyces paradoxus* D2-8 from the rhizosphere soil of reeds in Daqing soda saline-alkali land which exhibits saline-alkali tolerance, growth-promoting functions, and biocontrol activity. Pot and field experiments confirmed that this strain significantly promotes soybean growth, enhances soybean tolerance to soda saline-alkali stress, and increases yield. It was also found that the growth-promoting effect of this strain depends on the synthesis of substances such as indole-3-acetic acid (IAA), siderophores, and 1-aminocyclopropane-1-carboxylate (ACC) deaminase.

Although research on saline-alkali-tolerant and growth-promoting bacteria is relatively extensive, there remain issues such as insufficient exploration of strain resources, inadequate depth in functional mechanism analysis, and slow progress in the large-scale application of soybean saline-alkali-tolerant and growth-promoting bacteria. In this study, we systematically screened and identified an efficient saline-alkali-tolerant and growth-promoting bacterium, *Bacillus pumilus* AD14, from saline-alkali soil in Anda City, Heilongjiang Province. Through pot experiments with soybean, we verified that this strain can significantly enhance the saline-alkali tolerance of soybean and also has a beneficial effect on improving saline-alkali soil. Unlike many previous studies focusing on a single trait, strain AD14 uniquely integrates nitrogen fixation, phosphorus solubilization, and pectinase production under saline-alkali stress. More importantly, this study not only demonstrates enhanced plant stress tolerance but also reveals direct improvements in soil physicochemical properties, specifically reduced pH and soluble salt content, highlighting AD14’s dual role as both a plant biostimulant and a soil conditioner. Therefore, this work not only enriches the pool of saline-alkali-tolerant PGPB strains but also provides an excellent candidate for developing novel biological agents suitable for soybean cultivation in saline-alkali regions, holding important practical significance for promoting sustainable agricultural development in such areas.

## 2. Materials and Methods

### 2.1. Isolation and Purification of Bacterial Strains

The soil samples used in this study were collected from saline-alkali land in Anda City, Heilongjiang Province, China. The soil had a pH value of 8.44 and a soluble salt content of 1.18%. Bacterial strains were isolated from the saline-alkali soil samples using the dilution-plating method [14]. Specifically, 5 g of soil sample was dissolved in 45 mL of sterile water and shaken on a shaker at 180 r/min for 20 min, followed by standing. The supernatant was taken to obtain a 1 × 10^−1^ dilution. After gradient dilution with sterile water, a 1 × 10^−7^ dilution was achieved. The diluted solution was then spread onto LB solid medium and incubated at 37 °C for 4 days. Colonies with different morphologies were selected from the agar plates, and their morphological characteristics were observed. After three successive passages, each strain was transferred to LB liquid medium and shaken at 37 °C and 180 r/min for 12 h. The strains were preserved in sterile glycerol at a concentration of 50% and stored in an −80 °C freezer for future use.

### 2.2. Identification of Bacterial Strains

Morphological, physiological, biochemical, and molecular biological identifications were performed on the candidate strains. For morphological identification, the candidate strains were inoculated onto LB agar plates using the streaking method and incubated for 12–72 h, during which colony morphology was observed. Physiological and biochemical identification was carried out according to the methods described in the Manual for Identification of Common Bacterial Systems [15]. The isolated strains were subjected to Gram staining, catalase test, Voges–Proskauer (V-P) test, methyl red (M-R) test, and indole test. For molecular biological identification, genomic DNA of the bacterial strains was extracted using the EasyPure^®^ Bacterial Genomic DNA Kit (TransGen Biotech Co., Ltd., Beijing, China). Primers 27F (5′-GAGGTTTGATCGCGTCAG-3′) and 1541R (5′-GAGGTGTGATCGCA-3′) were selected for PCR amplification of the 16S rRNA gene. The PCR reaction was performed using the EasyTaq^®^ DNA Polymerase Kit (TransGen Biotech Co., Ltd., Beijing, China). The PCR products were detected by 1.0% agarose gel electrophoresis and sent to Comate Bioscience Co., Ltd. (Changchun, China) for sequencing. The sequencing results were analyzed using NCBI BLAST (https://blast.ncbi.nlm.nih.gov/Blast.cgi, accessed on 12 February 2024), and a phylogenetic tree was constructed using MEGA 7.0.

### 2.3. Analysis of Salt-Alkali Tolerance of Bacterial Strains

To evaluate the salt tolerance of the bacterial strains, they were inoculated onto LB solid media containing NaCl at concentrations of 4%, 6%, 8%, 10%, and 12%, respectively. For the alkali tolerance test, the strains were inoculated onto LB solid media with pH values adjusted to 8, 9, 10, 11, and 12 using NaOH. The growth of the strains was observed after 6 days of incubation at 37 °C. To assess the combined saline-alkali tolerance of the strains, four treatment groups were established based on the results of the individual salt and alkali tolerance tests (Treatment 1: 4% NaCl + pH 8; Treatment 2: 6% NaCl + pH 8; Treatment 3: 4% NaCl + pH 9; Treatment 4: 6% NaCl + pH 9). The strains were cultured under the same conditions as described above.

### 2.4. Analysis of the Growth Promotion and Hydrolase Production Ability of Strain

Based on the salt-alkali tolerance test results of 15 strains, the 6 strains AD1, AD8, AD10, AD11, AD13, and AD14 were selected for subsequent experiments. The nitrogen fixation, phosphorus solubilization, and potassium solubilization abilities of the six strains were tested using Ashby’s nitrogen-free medium, phosphate growth medium (NBRIP), and potassium solubilization medium [16]. After activation, single colonies were selected and inoculated, and the colony growth status was observed by dark cultivation at 37 °C for 7 days. Starch medium, skim milk medium, and pectin medium were used to detect the amylase, protease, and pectinase production abilities of the six strains [17]. The inoculation method of the strains was the same as above, and the growth status of the colonies was observed after 5 days of dark cultivation at 37 °C.

### 2.5. Analysis of the Soybean Growth-Promoting Effect of Strain

The strain was reactivated by inoculating the glycerol stock into LB liquid medium and cultured at 37 °C with shaking until the logarithmic phase, when the OD_600_ reached 0.8. The culture was then centrifuged at 4000 rpm for 15 min, and the bacterial cells were collected and diluted tenfold with sterile water (relative to the original culture volume) for subsequent use. The soybean variety used in this study was Dongnongdou 252, provided by the School of Life Sciences, Northeast Agricultural University. Plump and uniformly sized soybean seeds were selected and subjected to the following surface sterilization procedure: rinsing with sterile water for 15 min, treatment with 75% ethanol for 1 min, followed by five rinses with sterile water. The sterilized seeds were then placed in 9 cm diameter Petri dishes lined with double-layer sterile filter paper (10 seeds per dish). The experimental group received 4 mL of the diluted bacterial solution, while the control group received an equal volume of sterile water. The Petri dishes were incubated in a constant-temperature light incubator at 25 °C with a light intensity of 200 μmol·m^−2^·s^−1^. Each group was supplemented with the same volume of sterile water daily. Seed germination was considered effective when the embryonic root broke through the seed coat and reached a length of ≥2 mm [18]. The germination rate was calculated after 7 days. To evaluate the growth-promoting effect of the bacterial solution at the seedling stage, sterile vermiculite was used as the cultivation substrate, supplemented with 0.5× Hoagland nutrient solution as the nutritional source. Soybean seeds were sterilized as described above and sown in pots with a diameter of 15 cm (5 seeds per pot). The pots were maintained under a 16/8 h light/dark cycle at 25/22 °C (day/night), with a light intensity of 500 μmol·m^−2^·s^−1^. After 5 days of seed germination, the diluted bacterial solution was applied by watering the rhizosphere. The bacterial solution treatment was the same as described above, with 50 mL per pot, while the control group received an equal volume of sterile water. An appropriate amount of sterile water was added every 2 days. On the 30th day after seed germination, plant height, stem diameter, root number, root length, and root weight were measured and recorded [19].

### 2.6. Potted Experiment of Soybean in Saline-Alkali Soil with Bacillus pumilus AD14

The treatment method for soybean seeds and the AD14 bacterial solution was the same as described above, except that the cultivation substrate was replaced with natural saline-alkali soil provided by the Daqing Branch of the Heilongjiang Academy of Agricultural Sciences. Soil 1 had a pH of 7.82 and a soluble salt content of 0.73%, while Soil 2 had a pH of 8.44 and a soluble salt content of 1.12%. The sowing method, cultivation conditions, and bacterial solution application were the same as described above. On the 30th day after seed germination, the plant height, root number, root length, and root weight of soybean seedlings were measured and recorded. Leaf, root, and rhizosphere soil samples were collected from each group of soybean seedlings and immediately frozen in liquid nitrogen, then stored at −80 °C. Physiological indicators of soybean, including the activities of SOD, POD, and CAT, as well as the contents of malondialdehyde (MDA), soluble sugars, and soluble proteins, were determined using kits provided by Suzhou Grace Biotechnology Co., Ltd. (Suzhou, China). The kit models were as follows: superoxide dismutase (G0104F), peroxidase (G0108W), catalase (G0105W), malondialdehyde (G0110F), soluble sugar (G0501F), and soluble protein (G0417W). The operating procedures for the kits were obtained from the company’s website (https://www.geruisi-bio.com/, accessed on 12 September 2024). The pH of the rhizosphere soil was determined by potentiometry, and the soluble salt content was determined by the residue drying mass method [20]. Soil urease, sucrase, and catalase activities were determined using kit methods provided by Suzhou Grace Biotechnology Co., Ltd. (Suzhou, China). The kit models were as follows: soil urease (G0301W), soil sucrase (G0302W), and soil peroxidase (G0303W). The operating procedures for the kits were obtained from the company’s website (https://www.geruisi-bio.com/, accessed on 16 September 2024).

### 2.7. Data Statistics and Analysis

All experiments were performed with at least three independent biological replicates. For the germination rate experiment, each biological replicate consisted of 100 seeds per treatment (10 seeds in each of 10 Petri dishes). For the pot experiments, each biological replicate comprised 10 pots per treatment, with 5 seedlings per pot. Data from all biological replicates were pooled for statistical analysis.

## 3. Results

### 3.1. Isolation and Identification of Bacterial Strains

Fifteen bacterial strains were isolated and purified from saline-alkali soil using the dilution-plating method and were sequentially numbered AD1 to AD15. Observation and identification of the morphology of the 15 strains cultured at 37 °C revealed that the cell morphology of the strains was either rod-shaped or ovoid. The colony colors were milky white and light yellow. The strains exhibited relatively fast growth rates: 13 strains were observed to grow within 12 h, 1 strain within 24 h, and 1 strain within 72 h (Supplementary Figure S1, Table 1). The physiological and biochemical identification results showed that strains AD1, AD7, AD10, AD11, AD13, and AD14 were Gram-positive bacteria, while all other strains were Gram-negative. All strains except AD13 exhibited catalase activity. Strains AD2, AD6, AD10, AD11, AD13, AD14, AD15 tested negative in the Voges–Proskauer (V-P), methyl red (M-R), and indole tests, whereas the other strains tested positive (Table 2). The 16S rRNA genes of the 15 strains were amplified by PCR using the universal primers 27F and 1541R, and the PCR products were detected by agarose gel electrophoresis (Supplementary Figure S2). Comparison of the sequencing results with the NCBI database (Supplementary Figure S3) and the phylogenetic tree analysis showed that the 15 strains belonged to the following genera: *Enterobacter* (6 strains: AD3, AD4, AD5, AD8, AD9, AD12), *Bacillus* (5 strains: AD1, AD7, AD10, AD11, and *Bacillus pumilus* AD14), *Chryseobacterium* (1 strain: AD6), *Acinetobacter* (1 strain: AD15), *Enterococcus* (1 strain: AD13), and *Pseudomonas* (1 strain: AD2) (Supplementary Figure S4). In addition the 16S rDNA sequence of strain AD14 is provided in Appendix A.

### 3.2. Analysis of Salt and Alkali Tolerance of Bacterial Strains

The results of the saline-alkali tolerance test showed that the salt tolerance concentration range of the 15 strains was 4–10%, with an optimal growth concentration range of 4–6%. Among them, strain AD1 exhibited the strongest salt tolerance, tolerating a salt concentration of 10% (Table 3). The alkali stress tolerance test revealed that the 15 strains had a pH tolerance range of 8–11, with an optimal growth pH range of 8–9. Strain AD10 showed the strongest alkali tolerance and grew vigorously on pH 11 medium (Table 4). Based on the salt and alkali tolerance screening results, the optimal salt concentration range for the growth of the 15 strains was 4–6%, and the optimal pH range was 8–9. To further screen for strains with strong combined saline-alkali tolerance, a composite experiment was conducted using the optimal salt and alkali concentrations. The results showed that strains AD1, AD8, AD10, AD11, AD13, and AD14 exhibited strong saline-alkali tolerance (Table 5) and were therefore selected as candidate strains for subsequent experiments.

### 3.3. Analysis of the Growth-Promoting Ability of Bacterial Strains

The growth-promoting abilities of the six candidate saline-alkali-tolerant strains were evaluated. Among them, four strains—AD1, AD10, AD11, and AD14—exhibited nitrogen-fixing activity. Two strains, AD8 and AD14, showed phosphorus-solubilizing activity. No potassium-solubilizing activity was detected in any of the strains. Strains AD10, AD13, and AD14 possessed pectinase-producing ability, while no amylase or protease activity was detected (Table 6).

### 3.4. Analysis of the Promoting Effect of Bacterial Strains on Soybean Growth

**Figure 1:**
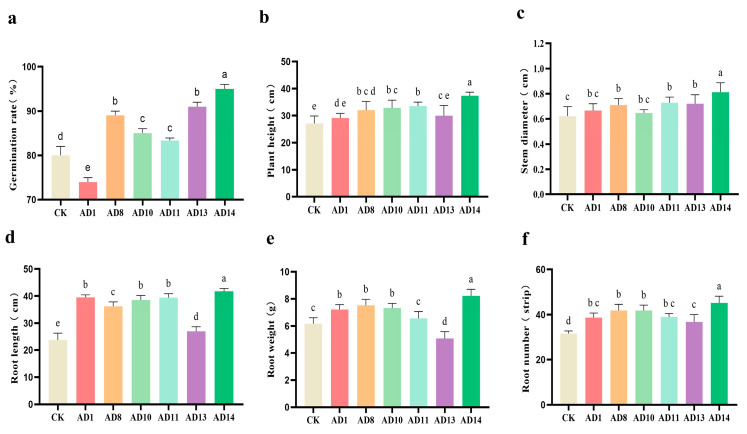
Effects of different bacterial strains on soybean germination and seedling growth. Note: (**a**) Germination rate (*n* = 100). (**b**) Plant height (cm, *n* = 10). (**c**) Stem diameter (cm, *n* = 10). (**d**) Root length (cm, *n* = 10). (**e**) Root weight (g, *n* = 10). (**f**) Root number per plant (*n* = 10). Different lowercase letters (a, b, c, d, e) above the bars indicate significant differences among different strain treatments (one-way ANOVA, *p* < 0.05).

The effects of the candidate strains AD1, AD8, AD10, AD11, AD13, and AD14 on soybean seed germination rate and seedling growth were tested. The results showed that after the use of the bacterial solution, five strains had varying degrees of promoting effect on soybean seed germination (Figure 1a); especially, the seed germination rate after treatment with strain AD14 was the highest, reaching 95%, which was 15% higher than that of the control group. In addition, one strain, AD1, was found to have inhibitory effects on soybean seed germination.

Overall, all six strains have a promoting effect on soybean seedling growth. From the perspective of individual morphological indicators, the application of bacterial solution significantly increased soybean plant height (Figure 1b), with strain AD14 showing the most prominent growth-promoting effect, increasing by 10.34 cm compared to the control group. Five strains increased the stem thickness of soybean seedlings (Figure 1c), with AD14 showing the best effect, increasing by 30.92% compared to the control group. All six strains can increase the root length of soybean seedlings (Figure 1d), among which strain AD14 has the best growth-promoting effect, with an increase of 76.48%. Five strains significantly increased the root weight of soybean seedlings (Figure 1e), among which strain AD14 had the best growth-promoting effect, with an increase of 2.042 g; six strains significantly increased the number of roots of soybean seedlings (Figure 1f), among which strain AD14 had the best growth-promoting effect, with an increase of 43.04%. Based on the above results, strain AD14 showed the best growth-promoting effect on soybeans among the candidate strains, so it was selected for subsequent experiments.

### 3.5. The Effect of Bacillus pumilus AD14 Liquid on Enhancing Soybean Salt-Alkali Tolerance

**Figure 2:**
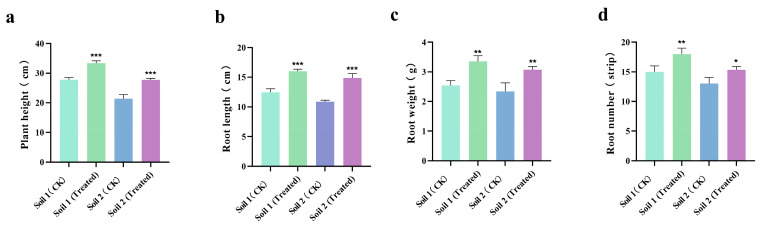
Effects of AD14 bacterial solution on soybean growth under saline-alkali stress. Note: (**a**) Plant height (cm). (**b**) Root length (cm). (**c**) Root weight (g). (**d**) Root number per plant. Asterisks indicate significant differences between AD14 treatment and the control group under the same soil condition (*, *p* < 0.05; **, *p* < 0.01; ***, *p* < 0.001; *n* = 10 per group).

Compared with the non-inoculated control, the growth indicators of soybean planted in two soils with different degrees of salinization were significantly improved following treatment with the AD14 bacterial solution. In terms of plant height, soybean plants grown in Soil 1 and Soil 2 increased by 5.63 cm and 6.37 cm, respectively, compared to the control group (Figure 2a). Regarding root length, soybean plants grown in the two soils showed increases of 3.58 cm and 3.99 cm, respectively, both of which were highly significant (Figure 2b). For root weight, the root weight of soybean plants grown in the two soils increased by 32.15% and 31.67%, respectively, compared to the control group, with a more pronounced effect observed in Soil 1 (Figure 2c). For root number, the root number of soybean plants grown in Soil 1 and Soil 2 increased significantly by 20.00% and 17.95%, respectively, compared to the control group (Figure 2d). Considering all four growth indicators, the application of the bacterial solution significantly improved the growth of soybean planted in both saline-alkali soils; however, the AD14 bacterial solution exhibited a better growth-promoting effect on soybean in Soil 1. This indicates that the bacterial solution promotes normal soybean growth under varying degrees of saline-alkali stress, particularly by reducing the inhibitory effect on plant growth in mildly saline-alkali soil and enhancing the resistance of soybean plants to saline-alkali stress.

**Figure 3:**
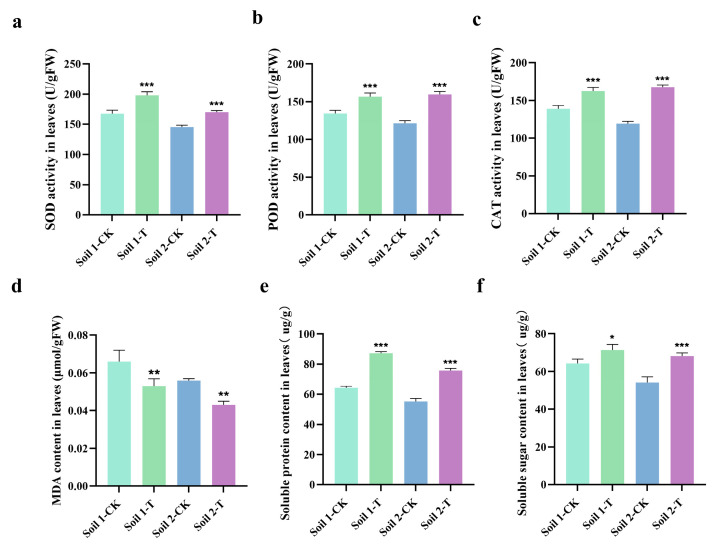
Effects of AD14 bacterial solution on the antioxidant system in soybean leaves under different saline-alkali soils. Note: (**a**) SOD activity (U/g FW). (**b**) POD activity (U/g FW). (**c**) CAT activity (U/g FW). (**d**) MDA content (nmol/g FW). (**e**) Soluble protein content (μg/g). (**f**) Soluble sugar content (μg/g). Asterisks indicate significant differences between AD14 treatment and the control group under the same soil condition (*, *p* < 0.05; **, *p* < 0.01; ***, *p* < 0.001; *n* = 10 per group).

After 30 days of planting, the application of the AD14 bacterial solution significantly increased the antioxidant enzyme activities in soybean leaves. For soybean plants grown in the two different soils, SOD activities in the leaves reached 198.23 U/g FW and 169.98 U/g FW, respectively, representing significant increases of 30.83 U/g FW and 24.75 U/g FW compared to the control group (Figure 3a). POD activities in the leaves reached 156.67 U/g FW and 159.67 U/g FW, respectively, showing significant increases of 22.5 U/g FW and 38.34 U/g FW relative to the control group (Figure 3b). CAT activities in the leaves reached 162.58 U/g FW and 167.68 U/g FW, respectively, with significant increases of 23.86 U/g FW and 48.45 U/g FW compared to the control group (Figure 3c). The MDA content in soybean leaves grown in the two different soils decreased by 19.70% and 23.21%, respectively, compared to the control group, and both decreases were statistically significant (Figure 3d). Regarding osmoregulatory substances, the soluble protein content in soybean leaves reached 87.33 μg/g and 75.76 μg/g, respectively, which were 35.90% and 37.17% higher than those in the control group (Figure 3e). The soluble sugar content reached 71.46 μg/g and 68.26 μg/g, respectively, representing increases of 11.19% and 25.87% compared to the control group. Among these, the effect on soybean leaves planted in Soil 2 was more pronounced (Figure 3f).

**Figure 4:**
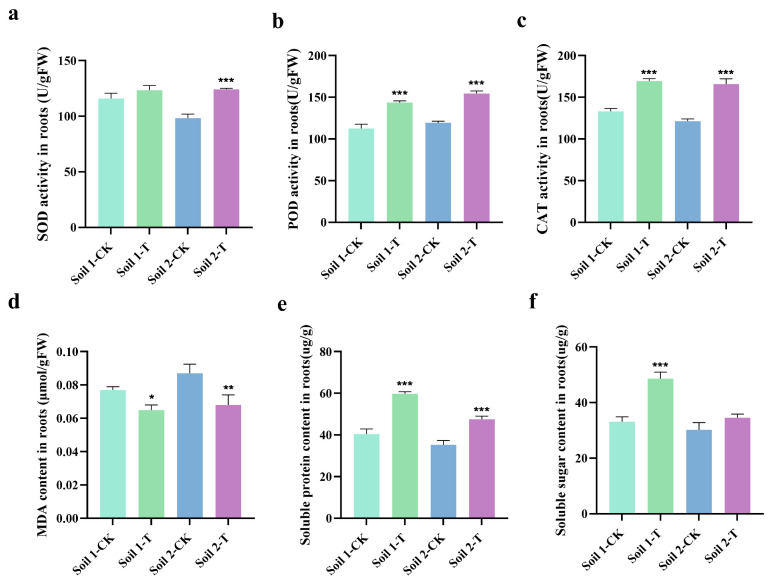
Effects of AD14 bacterial solution on the antioxidant system in soybean roots under different saline-alkali soils. Note: (**a**) SOD activity (U/g FW). (**b**) POD activity (U/g FW). (**c**) CAT activity (U/g FW). (**d**) MDA content (nmol/g FW). (**e**) Soluble protein content (μg/g). (**f**) Soluble sugar content (μg/g). Asterisks indicate significant differences between AD14 treatment and the control group under the same soil condition (*, *p* < 0.05; **, *p* < 0.01; ***, *p* < 0.001; *n* = 10 per group).

After 30 days of planting, application of the AD14 bacterial solution significantly increased the antioxidant enzyme activities in the root system of soybean plants. For soybean grown in the two different soils, SOD activities in the roots reached 123.46 U/g FW and 124.16 U/g FW, respectively, representing increases of 7.44 U/g FW and 25.82 U/g FW compared to the control group. Among these, the effect in the Soil 2 planting group was highly significant (Figure 4a). POD activities in the soybean roots reached 143.67 U/g FW and 154.50 U/g FW, respectively, showing significant increases of 31.04 U/g FW and 34.86 U/g FW relative to the control group (Figure 4b). CAT activities in the soybean roots reached 169.51 U/g FW and 165.99 U/g FW, respectively, with significant increases of 36.39 U/g FW and 44.65 U/g FW compared to the control group (Figure 4c). Compared with the control group, the MDA content in the roots of soybean grown in the two different soils decreased significantly by 15.58% and 21.84%, respectively, and the effect in the Soil 2 planting group was more pronounced (Figure 4d). Regarding osmoregulatory substances, the soluble protein content in soybean roots reached 59.77 μg/g and 47.58 μg/g, respectively, which were 47.87% and 34.72% higher than those in the control group (Figure 4e). The soluble sugar content reached 48.62 μg/g and 34.53 μg/g, respectively, representing increases of 46.80% and 14.22% compared to the control group (Figure 4f).

Based on the four growth indicators and six physiological and biochemical parameters described above, treatment with the AD14 bacterial solution significantly improved plant growth indicators, antioxidant enzyme activities, and osmoregulatory substance contents in soybean grown in both saline-alkali soils, accompanied by a decrease in MDA content. These results indicate that the AD14 bacterial solution reduces oxidative damage by activating the antioxidant defense system of soybean. Concurrently, it promotes the accumulation of osmoregulatory substances and helps to maintain cellular homeostasis. This dual regulatory mechanism may represent an important physiological basis by which this strain effectively alleviates saline-alkali stress and promotes soybean growth.

### 3.6. The Effect of Bacillus pumilus AD14 Liquid on the Physicochemical Properties and Enzyme Activity of Saline-Alkali Soil

**Figure 5:**
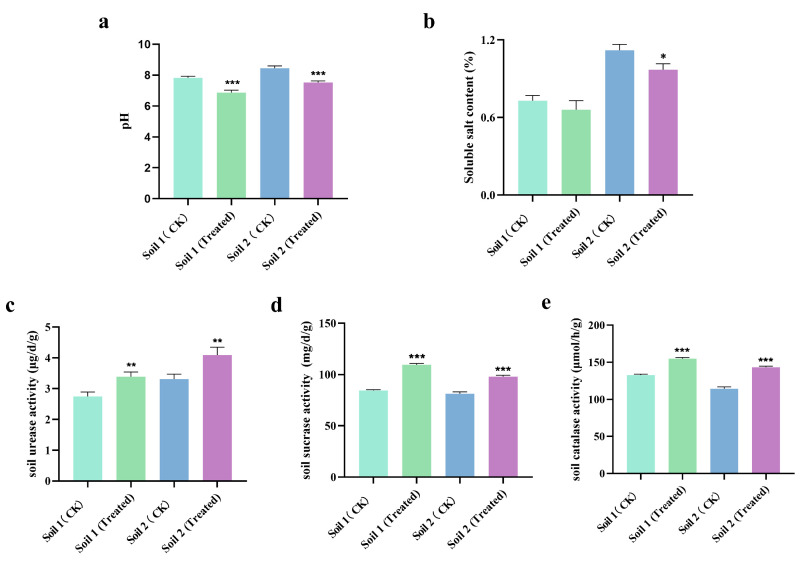
Effects of AD14 bacterial solution on physicochemical properties of different saline-alkali soils. Note: (**a**) Soil pH value. (**b**) Soluble salt content (%). (**c**) Urease activity (μg/d/g). (**d**) Sucrase activity (mg/d/g). (**e**) Catalase activity (μmol/h/g). Asterisks indicate significant differences between AD14 treatment and the control group under the same soil condition (*, *p* < 0.05; **, *p* < 0.01; ***, *p* < 0.001; *n* = 6 per group).

Analysis of the physicochemical properties of the saline-alkali soil after the pot experiment revealed that the pH values in the AD14 bacterial solution treatment groups were 6.87 and 7.52, respectively, representing significant reductions of 12.15% and 10.94% compared to the control group (Figure 5a). The soluble salt contents were 0.66% and 0.97%, respectively, corresponding to decreases of 9.59% and 13.39% relative to the control group (Figure 5b). The bacterial solution treatment exhibited a significant ameliorative effect on Soil 2.

Measurement of soil enzyme activities after the pot experiment showed that the activities of urease, sucrase, and catalase in the AD14 bacterial solution treatment groups were significantly increased in both saline-alkali soils compared to the control group. Urease activities reached 3.39 μg/d/g and 4.09 μg/d/g, respectively, representing increases of 23.42% and 23.56% relative to the control group (Figure 5c). Sucrase activities reached 109.58 mg/d/g and 98.06 mg/d/g, respectively, showing increases of 25.10 mg/d/g and 16.72 mg/d/g compared to the control group (Figure 5d). Catalase activities reached 154.93 μmol/h/g and 143.32 μmol/h/g, respectively, with increases of 22.11 μmol/h/g and 28.99 μmol/h/g relative to the control group (Figure 5e). Based on the five indicators described above, inoculation with the AD14 bacterial solution significantly improved the physicochemical properties and enzymatic characteristics of both saline-alkali soils, as manifested by a marked decrease in soil pH and soluble salt content, as well as a significant increase in urease, sucrase, and catalase activities. These results indicate that the AD14 bacterial solution directly alleviates saline-alkali stress damage to plants by reducing soil alkalinity and salt content. Furthermore, it improves soil fertility and rhizosphere microenvironment health by activating the soil nitrogen cycle, carbon cycle, and antioxidant detoxification system.

## 4. Discussion

### 4.1. Isolation and Screening of Saline-Alkali-Tolerant Plant Growth-Promoting Bacteria

The global issue of salinization poses a severe challenge to agricultural production. Soil salinization can cause ion toxicity, hypoxia, oxidative damage, and overall inhibition of plant growth and development, ultimately leading to reduced yields and even plant death [21]. In northeastern China, this problem is particularly prominent, and soil salinization has become the main limiting factor for the cultivation of important crops such as soybean [22]. Improving saline-alkali soil and enhancing crop saline-alkali tolerance are key to addressing this challenge, and saline-alkali-tolerant PGPB holds great potential for application in saline-alkali land improvement [23]. For example, Dharman Sridhar et al. [24] screened and identified three salt-tolerant and growth-promoting strains (*Teudomonas toyotomisis*, *Bacillus subtilis*, and *Bacillus cereus*) from saline-alkali soil. Inoculation of these strains into saline-alkali soil used for sesame cultivation resulted in significant improvements in sesame oil quality and soil physicochemical properties. Wang M et al. [25] applied nine selected growth-promoting strains to blueberry planting soil and found that branch number, leaf number, plant height, and chlorophyll content in the treatment group were significantly higher than those in the control group. Nishtha R. Vaghella et al. [26] isolated three salt-tolerant and growth-promoting bacterial strains (*Streptomyces* sp. KhEc 44, *Bacillus paralicheniformis* KhEc 68, and *Priestia filamentosa* KhEc 69) from the rhizosphere soil of *Euphorbia caducifolia* L. grown in saline-alkali soil and found that these strains increased mung bean yield under saline-alkali field conditions. Based on these findings, the present study took saline-alkali soil from Anda City, Heilongjiang Province, as the research object, screened saline-alkali-tolerant and growth-promoting strains, and provided new ideas for solving local soybean planting problems.

Compared with previous studies, the innovation of this research lies in the unique combination of multiple growth-promoting traits (nitrogen fixation, phosphorus solubilization, and pectinase production) and, more importantly, the dual plant–soil improvement effect demonstrated under pot conditions an integrated function that has rarely been systematically reported for this species in saline-alkali soybean cultivation. This strain exhibits strong saline-alkali tolerance, growing vigorously in environments with a salt concentration of 8% and a pH of 9 (Table 3 and Table 4), demonstrating its superior survival ability in saline-alkali soils. Compared with other common saline-alkali-tolerant and growth-promoting strains, AD14 shows significant advantages in multiple plant growth-promoting functions, including nitrogen fixation and phosphorus solubilization, which can provide additional nitrogen and phosphorus sources for soybean and promote its growth. Notably, in saline-alkali soil, AD14 not only possesses good growth-promoting ability but also secretes extracellular enzymes such as pectinase, which improve soil structure, enhance soil fertility, and increase soil enzyme activity (Table 6). This strain has demonstrated unique advantages in enhancing saline-alkali resistance in soybean, providing new strain resources and application directions for improving soil salinization and facilitating soybean cultivation in saline-alkali soils.

### 4.2. Effect of Bacillus pumilus AD14 Liquid on Salt-Alkali Tolerance of Soybean Plants

Under saline-alkali stress, soybeans face the dual challenges of oxidative damage and osmotic imbalance. The antioxidant system and osmotic regulators work together to ensure their growth [27]. The antioxidant system, composed of enzymes such as SOD, POD, and CAT as well as non-enzymatic substances, gradually scavenges excess reactive oxygen species (ROS), reduces MDA accumulation, protects the integrity of cell membranes and organelles, and maintains normal metabolic processes such as photosynthesis and respiration [28]. Osmotic substances (e.g., soluble sugars and soluble proteins) enhance cellular osmotic pressure, alleviate physiological drought, reduce water loss and ion toxicity, and ensure nutrient absorption and transport [29]. The synergistic effect of these two mechanisms effectively alleviates stress inhibition, promotes dry matter accumulation and yield increase in soybean, significantly improves its adaptability to saline-alkali environments, and provides important physiological support for soybean cultivation in saline-alkali lands.

The present study found that application of *Bacillus pumilus* AD14 bacterial solution significantly improved the growth and saline-alkali resistance of soybean in saline-alkali soils. Notably, in mildly saline-alkali soil, soybean plants treated with the bacterial solution exhibited significant improvements in growth indicators such as plant height, root length, root number, and root weight (Figure 2). The application of the bacterial solution not only improved the growth morphology of soybean but also enhanced its ability to withstand saline-alkali stress, as manifested by increased antioxidant enzyme activities in leaves and roots. After bacterial solution application, the activities of antioxidant enzymes (SOD, POD, and CAT) in soybean leaves and roots were significantly increased, while MDA content was significantly reduced (Figure 3 and Figure 4). In particular, in moderately saline-alkali soil, the activities of SOD, POD, and CAT in the soybean root system increased by 26.26%, 29.13%, and 36.80%, respectively. Antioxidant enzymes are key enzymes for plants to cope with saline-alkali stress, as they effectively scavenge ROS induced by saline-alkali stress and protect plant cells from oxidative damage [30]. Therefore, the application of the bacterial solution enhances the stress resistance of soybean by increasing antioxidant enzyme activities, which is beneficial for soybean growth in saline-alkali environments.

In addition, bacterial solution treatment significantly increased the content of soluble sugars and soluble proteins in soybean leaves and roots. In moderately saline-alkali soil, the increases in soluble sugars and soluble proteins in leaves reached 25.87% and 37.17%, respectively (Figure 3e,f). These osmotic substances help to maintain intracellular water balance and alleviate the effects of saline-alkali stress on plants [31]. This indicates that strain AD14 not only promotes soybean growth but also enhances its adaptability to saline-alkali stress.

Overall, *Bacillus pumilus* AD14 enhances the stress resistance of soybean in saline-alkali environments by increasing antioxidant enzyme activities and osmotic substance contents. These results demonstrate that strain AD14 significantly improves the saline-alkali tolerance of soybean in saline-alkali soils, effectively alleviating the adverse effects of such soils on soybean growth. We acknowledge several limitations in this study. First, the results are currently limited to soybean pot experiments, which differ from complex field conditions. Factors such as environmental fluctuations and competition from indigenous microorganisms may affect the colonization and performance of strain AD14, and its field efficacy has not yet been validated. In addition, the long-term ecological impact of this strain on saline-alkali soil remains unknown. Second, the lack of a sterilized inoculum control (heat-killed bacteria) limits our ability to attribute the observed effects solely to the biological activity of live AD14 cells. Furthermore, a comparison with commercial PGPB strains, while valuable, is beyond the current scope. Future research should include field trials, long-term risk monitoring, killed-cell controls, and commercial strain comparisons to support the large-scale application of AD14.

### 4.3. Improvement Effect of Bacillus pumilus AD14 Liquid on Saline-Alkali Soil for Soybean Cultivation

In the context of saline-alkali land improvement, the application of saline-alkali-tolerant growth-promoting bacterial solutions has been proven to be an effective approach for improving soil physicochemical properties, enhancing soil fertility, and increasing microbial activity [32]. Wenxiao Cui et al. [33] isolated *Penicillium kloeckeri* from the soybean rhizosphere in soybean-growing areas of Heilongjiang Province and inoculated it into saline-alkali soil. The results showed a decrease in soil pH, an increase in organic matter content, and enhanced soil enzyme activity. Alshaal et al. [34] inoculated three saline-alkali-tolerant and growth-promoting bacterial strains (*Azospirillum brasilense* SARS 1001, *Bacillus circulans* NCAIM B.02324, and *Pseudomonas koreensis* MG209738) into saline-alkali soil for sunflower cultivation and observed a decrease in soil electrical conductivity. Jing et al. [35] mixed *Bacillus flexus* with biochar into the soil of Bauhinia seedlings and found that this treatment reduced soil pH and electrical conductivity. Consistent with these findings, the present study showed that application of *Bacillus pumilus* AD14 bacterial solution significantly reduced the pH value and salt content of saline-alkali soil. The improvement effect was particularly prominent in moderately saline-alkali soil, with decreases of 10.94% in pH and 13.39% in salt content, respectively (Figure 5a,b). Multiple studies have confirmed that the application of PGPB can effectively improve soil physicochemical properties and enhance soil enzyme activity. Such improvement effects are often closely associated with the synergistic effects of multiple bacterial functions, including nitrogen fixation, phosphorus solubilization, and hydrolase production [36,37,38]. The present study further found that strain AD14 significantly enhanced soil enzyme activities (Figure 5c–e). The most pronounced improvement effects were observed on urease and sucrase activities in mildly saline-alkali soil, with increases of 23.42% and 29.71%, respectively. These results not only clarify the application value of strain AD14 in saline-alkali land improvement but also provide specific support for its mechanism of promoting crop growth by optimizing soil fertility.

## 5. Conclusions

In this study, 15 salt-tolerant bacterial strains were screened and identified from saline-alkali soil in Anda City, Heilongjiang Province. Through a series of saline-alkali tolerance tests and functional analyses, *Bacillus pumilus* AD14 was selected as the primary saline-alkali-tolerant and growth-promoting strain. This strain not only exhibits strong saline-alkali tolerance and multiple plant growth-promoting functions, including nitrogen fixation, phosphorus solubilization, and pectinase production, but also significantly promotes soybean growth and enhances soybean resistance to saline-alkali stress, as demonstrated in pot experiments. Furthermore, this strain significantly reduces soil pH and salt content while substantially increasing soil enzyme activities. Collectively, these findings indicate that Bacillus pumilus AD14 provides a multifunctional and dual-effect solution, enhancing crop saline-alkali tolerance while simultaneously ameliorating soil properties, offering a novel microbial strategy distinct from single-trait or single-effect PGPB strains.

## 6. Patents

*Bacillus pumilus* AD14 has been granted a Chinese national invention patent, with authorization announcement number CN 118652689 B.

## Figures and Tables

**Table 1:** Morphological characteristics of 15 strains.

Strain	Cell Morphology	Colony Characteristics (LB Medium)	Growth Rate
AD1	straight rod	Colony milky white with smooth edges and central bulge	* rapid
AD2	short rod	Colony milky white with irregular edges and no central bulge	* rapid
AD3	short rod	Colony milky white with smooth edges and central bulge	* rapid
AD4	short rod	Colony pale yellow with irregular edges and slightly raised center	* rapid
AD5	short rod	Colony pale yellow with smooth edges and central bulge	* rapid
AD6	thick rod	Colony pale yellow with smooth edges and slightly raised center	* rapid
AD7	straight rod	Colony milky white with smooth edges and central bulge	* rapid
AD8	short rod	Colony milky white with smooth edges and central bulge	*** slow
AD9	short rod	Colony milky white with irregular edges and slightly raised center	* rapid
AD10	straight rod	Colony milky white with irregular edges and slightly raised center	* rapid
AD11	straight rod	Colony pale yellow with smooth edges and central bulge	* rapid
AD12	short rod	Colony milky white with irregular edges and slightly raised center	* rapid
AD13	oval	Colony pale yellow with smooth edges and central bulge	* rapid
AD14	straight rod	Colony milky white with smooth edges and slightly raised center	* rapid
AD15	coccoid rod	Colony milky white with irregular edges and slightly raised center	** moderate

Note: “*” Growth observed at 12 h (rapid); “**” growth observed at 24 h (moderate); “***” growth observed at 72 h (slow).

**Table 2:** Some physiological and biochemical parameters of 15 strains.

Strain	Physiological and Biochemical Indicators				
	Gram Stain	Contact Enzyme Experiment	V-P Test	M-R Test	Indole Test
AD1	+	+	+	+	+
AD2	−	+	−	−	−
AD3	−	+	+	+	+
AD4	−	+	+	+	+
AD5	−	+	+	+	+
AD6	−	+	−	−	−
AD7	+	+	+	+	+
AD8	−	+	+	+	+
AD9	−	+	+	+	+
AD10	+	+	−	−	−
AD11	+	+	−	−	−
AD12	−	+	+	+	+
AD13	+	−	−	−	−
AD14	+	+	−	−	−
AD15	−	+	−	−	−

Note: “+” positive; “−” negative.

**Table 3:** The growth of strains on plates with different salt concentrations.

Strain	Salt Concentration (%)				
	4%	6%	8%	10%	12%
AD1	+++	+++	+++	++	−
AD2	+	+	−	−	−
AD3	++	++	−	−	−
AD4	+++	+++	−	−	−
AD5	+++	+++	−	−	−
AD6	+	+	−	−	−
AD7	+++	+++	++	−	−
AD8	+++	+++	−	−	−
AD9	+++	+++	−	−	−
AD10	+++	+++	−	−	−
AD11	+	+	−	−	−
AD12	+++	+++	+++	−	−
AD13	+++	+++	+++	−	−
AD14	+++	+++	+++	−	−
AD15	+++	+++	−	−	−

Note: “+++” means vigorous growth of the colony on the medium; “++” means more growth of the colony on the medium; “+” means less growth of the colony on the medium; “−” means no growth of the colony on the medium.

**Table 4:** The growth of strains on plates with different alkali concentrations.

Strain	pH Value				
	8	9	10	11	12
AD1	+++	+++	−	−	−
AD2	+	−	−	−	−
AD3	+	−	−	−	−
AD4	+++	+++	++	+	−
AD5	+++	+++	−	−	−
AD6	+	−	−	−	−
AD7	++	++	+	−	−
AD8	+++	+++	+	+	−
AD9	+++	+++	−	−	−
AD10	+++	+++	+++	+++	−
AD11	+++	+++	++	++	−
AD12	+++	+++	−	−	−
AD13	+++	+++	++	++	−
AD14	+++	+++	−	−	−
AD15	+++	+++	−	−	−

Note: “+++” means vigorous growth of the colony on the medium; “++” means more growth of the colony on the medium; “+” means less growth of the colony on the medium; “−” means no growth of the colony on the medium.

**Table 5:** Growth of the strain on plates under optimal salt and alkaline conditions.

Strain	Treatment 1	Treatment 2	Treatment 3	Treatment 4
AD1	+++	+++	++	++
AD2	+	−	−	−
AD3	−	−	−	−
AD4	+++	−	−	−
AD5	+++	+++	−	−
AD6	+	−	−	−
AD7	+++	+++	−	−
AD8	+++	+++	+++	+++
AD9	+++	−	++	−
AD10	+++	+++	+++	+++
AD11	+++	+++	+++	+++
AD12	+++	+++	−	−
AD13	+++	++	++	+
AD14	+++	+++	++	+
AD15	+++	+++	++	−

Note: “+++” means vigorous growth of the colony on the medium; “++” means more growth of the colony on the medium; “+” means less growth of the colony on the medium; “−” means no growth of the colony on the medium.

**Table 6:** Results of identification of growth-promoting function of strain.

Strain	Nitrogen Fixation	Dissolved Phosphorus	Potassium Solution	Pectinase	Amylase	Proteinogenesis
AD1	+	−	−	−	−	−
AD8	−	+	−	−	−	−
AD10	+	−	−	+	−	−
AD11	+	−	−	−	−	−
AD13	−	−	−	+	−	−
AD14	+	+	−	+	−	−

Note: “+” indicates that the strain has this function; “−” indicates that the strain does not have this function.

## Data Availability

The original contributions presented in this study have been included in the article. For further inquiries, please contact the corresponding author.

## Supplementary Materials

The following supporting information can be downloaded at: https://www.mdpi.com/article/10.3390/microorganisms14061168/s1, Figure S1: Colony morphology of 15 saline-alkali-tolerant strains. Figure S2: Agarose gel electrophoresis of PCR amplification products from 15 bacterial strains. Note: M: DNA molecular weight standard 2000; 1–15: Colony PCR products. Figure S3: NCBI BLAST Analysis of 15 Isolates. Note: a–o are the comparison results of AD1–15. Figure S4: Phylogenetic tree of strain AD14 based on 16S rDNA sequence. We confirm that these are the correct captions for all Supplementary Materials.

## Abbreviations

The following abbreviations are used in this manuscript:

LB	Lysogeny broth
PGPB	Plant growth-promoting bacteria
FAO	Food and Agriculture Organization
SOD	Superoxide Dismutase
POD	Peroxidase
CAT	Catalase
MDA	Malondialdehyde
IAA	Indole-3-Acetic Acid
V-P	Voges–Proskauer
M-R	Methyl red
ACCD	1-aminocyclopropane-1-carboxylate deaminase
ROS	Reactive oxygen species

## Appendix A

### Bacillus pumilus AD14 Based on the Sequence of 16S rDNA

TTGCAGGGCGGGTGCTATACATGCAGTCGAGCGGACAGAAGGGAGCTTGCTCCCGGATGTTAGCGGCGGACGGGTGAGTAACACGTGGGTAACCTGCCTGTAAGACTGGGATAACTCCGGGAAACCGGAGCTAATACCGGATAGTTCCTTGAACCGCATGGTTCAAGGATGAAAGACGGTTTCGGCTGTCACTTACAGATGGACCCGCGGCGCATTAGCTAGTTGGTGGGGTAATGGCTCACCAAGGCGACGATGCGTAGCCGACCTGAGAGGGTGATCGGCCACACTGGGACTGAGACACGGCCCAGACTCCTACGGGAGGCAGCAGTAGGGAATCTTCCGCAATGGACGAAAGTCTGACGGAGCAACGCCGCGTGAGTGATGAAGGTTTTCGGATCGTAAAGCTCTGTTGTTAGGGAAGAACAAGTGCGAGAGTAACTGCTCGCACCTTGACGGTACCTAACCAGAAAGCCACGGCTAACTACGTGCCAGCAGCCGCGGTAATACGTAGGTGGCAAGCGTTGTCCGGAATTATTGGGCGTAAAGGGCTCGCAGGCGGTTTCTTAAGTCTGATGTGAAAGCCCCCGGCTCAACCGGGGAGGGTCATTGGAAACTGGGAAACTTGAGTGCAGAAGAGGAGAGTGGAATTCCACGTGTAGCGGTGAAATGCGTAGAGATGTGGAGGAACACCAGTGGCGAAGGCGACTCTCTGGTCTGTAACTGACGCTGAGGAGCGAAAGCGTGGGGAGCGAACAGGATTAGATACCCTGGTAGTCCACGCCGTAAACGATGAGTGCTAAGTGTTAGGGGGTTTCCGCCCCTTAGTGCTGCAGCTAACGCATTAAGCACTCCGCCTGGGGAGTACGGTCGCAAGACTGAAACTCAAGGAATTGACGGGGGCCCGCACAAGCGGTGGAGCATGTGGTTTAATTCGAAGCAACGCGAAGAACCTTACCAGTCTGACATCTTCTGACACCCTAAAGATAGGGCTTCCCTTCGGGGACGAGTGACAGTGTGCATGTTGTCGTCA

## References

[1] Litalien A., Zeeb B. (2020). Curing the earth: A review of anthropogenic soil salinization and plant-based strategies for sustainable mitigation. Sci. Total Environ..

[2] Jin S., Wang X., Dong Y., Li G., Chang X., Zhang L. (2022). The gene LpBCP increased NaHCO_3_ resistance by enhancing lignin or ROS scavenging in the Nicotiana benthamiana. Plant Biol..

[3] Vianna G.R., Cunha N.B., Rech E.L. (2023). Soybean seed protein storage vacuoles for expression of recombinant molecules. Curr. Opin. Plant Biol..

[4] Zhang Y., Ding J., Wang H., Su L., Zhao C. (2020). Biochar addition alleviate the negative effects of drought and salinity stress on soybean productivity and water use efficiency. BMC Plant Biol..

[5] Khan M., Ali S., Al Azzawi T.N.I., Saqib S., Ullah F., Ayaz A., Zaman W. (2023). The Key Roles of ROS and RNS as a Signaling Molecule in Plant-Microbe Interactions. Antioxidants.

[6] Huang W., Tu J., Ya J., Zhang Q., Liu Y., Zheng D., Rao G., Xue Y. (2025). Comparative transcriptome analysis reveals the complex response mechanisms of soybean leaves and roots to salt stress. BMC Plant Biol..

[7] Heng T., He X.L., Yang L.L., Xu X., Feng Y. (2022). Mechanism of Saline-Alkali land improvement using subsurface pipe and vertical well drainage measures and its response to agricultural soil ecosystem. Environ. Pollut..

[8] Zhao H.L., Yu J.Y., Liu T., Wang L., Zhao Y. (2023). Application of Desulphurized Gypsum with Straw to Improve Physicochemical Properties of Saline-alkali Land in Yellow River Delta. Huan Jing Ke Xue.

[9] Song J., Guan X., Cui H., Liu L., Li Y., Li Y., Ma S. (2025). The impact of salt-tolerant plants on soil nutrients and microbial communities in soda saline-alkali lands of the Songnen plain. Front. Microbiol..

[10] Arminjon L., Lefort F. (2025). Quick In Vitro Screening of PGPMs for Salt Tolerance and Evaluation of Induced Tolerance to Saline Stress in Tomato Culture. Microorganisms.

[11] Shi M., Zhang L., Sun H., Ji S., Cui H., Wan W., Liu X., Tian A., Yang W., Wang X. (2025). The Plant Growth-Promoting *Bacterium Bacillus cereus* LpBc-47 Can Alleviate the Damage of Saline-Alkali Stress to Lilium pumilum. Microorganisms.

[12] Li G., Shi M., Wan W., Wang Z., Ji S., Yang F., Jin S., Zhang J. (2024). Maize Endophytic Plant Growth-Promoting Bacteria *Peribacillus simplex* Can Alleviate Plant Saline and Alkaline Stress. Int. J. Mol. Sci..

[13] Gao Y., Han Y., Li X., Li M., Wang C., Li Z., Wang Y., Wang W. (2022). A Salt-Tolerant Streptomyces paradoxus D2-8 from Rhizosphere Soil of Phragmites communis Augments Soybean Tolerance to Soda Saline-Alkali Stress. Pol. J. Microbiol..

[14] Liu H., Hu L., Wang X., You X., Jiang H., Hou S., Li J., Liu K. (2025). High-throughput cultivation and screening of plant-promoting bacteria with nitrogen fixation from mangrove sediments. J. Appl. Microbiol..

[15] Awong-Taylor J., Craven K.S., Griffiths L., Bass C., Muscarella M. (2008). Comparison of biochemical and molecular methods for the identification of bacterial isolates associated with failed loggerhead sea turtle eggs. J. Appl. Microbiol..

[16] Sultana S., Paul S.C., Parveen S., Alam S., Rahman N., Jannat B., Hoque S., Rahman M.T., Karim M.M. (2020). Isolation and identification of salt-tolerant plant-growth-promoting rhizobacteria and their application for rice cultivation under salt stress. Can. J. Microbiol..

[17] Mukhtar S., Zareen M., Khaliq Z., Mehnaz S., Malik K.A. (2020). Phylogenetic analysis of halophyte-associated rhizobacteria and effect of halotolerant and halophilic phosphate-solubilizing biofertilizers on maize growth under salinity stress conditions. J. Appl. Microbiol..

[18] Wong W.T., Tseng C.H., Hsu S.H., Lur H.-S., Mo C.-W., Huang C.-N., Hsu S.-C., Lee K.-T., Liu C.-T. (2014). Promoting effects of a single *Rhodopseudomonas palustris* inoculant on plant growth by Brassica rapa chinensis under low fertilizer input. Microbes Environ..

[19] Feng L., Raza M.A., Li Z., Chen Y., Bin Khalid M.H., Du J., Liu W., Wu X., Song C., Yu L. (2019). The Influence of Light Intensity and Leaf Movement on Photosynthesis Characteristics and Carbon Balance of Soybean. Front. Plant Sci..

[20] Hardie M., Doyle R. (2012). Measuring soil salinity. Methods Mol. Biol..

[21] Yi E., Zhang J., Jiang Y., Yang L., Sun M., Wang L., Li X., Li Y., Dong Y., Ma L. (2025). *Bacillus amyloliquefaciens* YB1701 Enhances Rice Tolerance to Saline-Alkali Stress Via ROS Scavenging and Ion Homeostasis. Curr. Microbiol..

[22] Wang Y., Zhang M., Lu L., Wang C., Wang J., Hu Y., Li S., Xie W., Hu X., Guo H. (2025). Effects of rot-promoting bacteria on decomposition characteristics of corn straw and spring soybean yield in Saline-alkali Land. Front. Plant Sci..

[23] Lu L., Liu N., Fan Z., Liu M., Zhang X., Tian J., Yu Y., Lin H., Huang Y., Kong Z. (2024). A novel PGPR strain, *Streptomyces lasalocidi* JCM 3373T, alleviates salt stress and shapes root architecture in soybean by secreting indole-3-carboxaldehyde. Plant Cell Environ..

[24] Sridhar D., Alherwairini S.S., Eswaran S.U.D., Barasarathi J., Lalitha S., Sayyed R. (2025). The soil microbiome enhances sesame growth and oil composition, and soil nutrients under saline conditions. Sci. Rep..

[25] Wang M., Yang X. (2024). Effects of plant growth-promoting rhizobacteria on blueberry growth and rhizosphere soil microenvironment. PeerJ.

[26] Vaghela N.R., Gohel S.D. (2025). Liquid formulation of halo-alkali-thermo-tolerant rhizobacteria for enhanced growth of mung bean crops under abiotic stresses. Sci. Rep..

[27] Yang F., Lv G. (2025). Responses of Calligonum leucocladum to Prolonged Drought Stress Through Antioxidant System Activation, Soluble Sugar Accumulation, and Maintaining Photosynthetic Homeostasis. Int. J. Mol. Sci..

[28] Zhao S., Liang T., Zhou T., Li D., Wang B., Zhan J., Liu L. (2018). Biotransformation and responses of antioxidant enzymes in hydroponically cultured soybean and pumpkin exposed to perfluorooctane sulfonamide (FOSA). Ecotoxicol. Environ. Saf..

[29] Feng D., Liu W., Chen K., Ning S., Gao Q., Chen J., Liu J., Sun X., Xu W. (2024). Exogenous Substances Used to Relieve Plants from Drought Stress and Their Associated Underlying Mechanisms. Int. J. Mol. Sci..

[30] Wang F., Song N. (2019). Salinity-induced alterations in plant growth, antioxidant enzyme activities, and lead transportation and accumulation in Suaeda salsa: Implications for phytoremediation. Ecotoxicology.

[31] Tan Y., Liang Z., Shao H., Du F. (2006). Effect of water deficits on the activity of anti-oxidative enzymes and osmoregulation among three different genotypes of Radix Astragali at seeding stage. Colloids Surf. B Biointerfaces.

[32] Liu B., Jia P., Zou J., Ren H., Xi M., Jiang Z. (2025). Improving soil properties and Sesbania growth through combined organic amendment strategies in a coastal saline-alkali soil. J. Environ. Manag..

[33] Cui W., Wu Y., Ni B., Cao J. (2025). Whole genome sequence of *Penicillium kloeckeri* and insight into its growth-promoting, saline alkaline tolerance properties. Front. Microbiol..

[34] Alshaal T., Alharbi K., Naif E., Rashwan E., Omara A.E.-D., Hafez E.M. (2024). Strengthen sunflowers resilience to cadmium in saline-alkali soil by PGPR-augmented biochar. Ecotoxicol. Environ. Saf..

[35] Jing D., Liu F., Liu B., Peng L., Sun M., Ma H., Du Z. (2025). Effects of Biochar and PGPR Application on the Physicochemical Properties and Humus Components of Soil Used for Planting Fruit Mulberry Seedlings Under Salt Stress. Biology.

[36] Siebielec S., Woźniak M.M., Nowak A., Siebielec G., Kozieł M., Sugier P., Jaroszuk-Ściseł J. (2025). Plant growth promotion mechanisms of bacteria isolated from a long-term reclaimed smelter waste deposit. Sci. Rep..

[37] He D., Wan W. (2022). Distribution of Culturable Phosphate-Solubilizing Bacteria in Soil Aggregates and Their Potential for Phosphorus Acquisition. Microbiol. Spectr..

[38] Alswat A.S., Alharthy O.M., Alzahrani S.S., Alhelaify S.S. (2024). Halophilic Pectinase-Producing Bacteria from Arthrocnemum macrostachyum Rhizosphere: Potential for Fruit-Vegetable Juice Processing. Microorganisms.

