# Supplementary material for: Bacillus pumilus AD14: A Saline-Alkali-Tolerant Plant Growth-Promoting Bacterium for Enhancing Soybean Tolerance and Ameliorating Saline-Alkali Soil
Source: Microorganisms. 2026 May 22;14(6):1168. doi: 10.3390/microorganisms14061168 (PMC13303088; doi:10.3390/microorganisms14061168)
Supplement: Supplementary file 1 [file microorganisms-14-01168-s001.zip › microorganisms-4294781-supplementary.pdf]

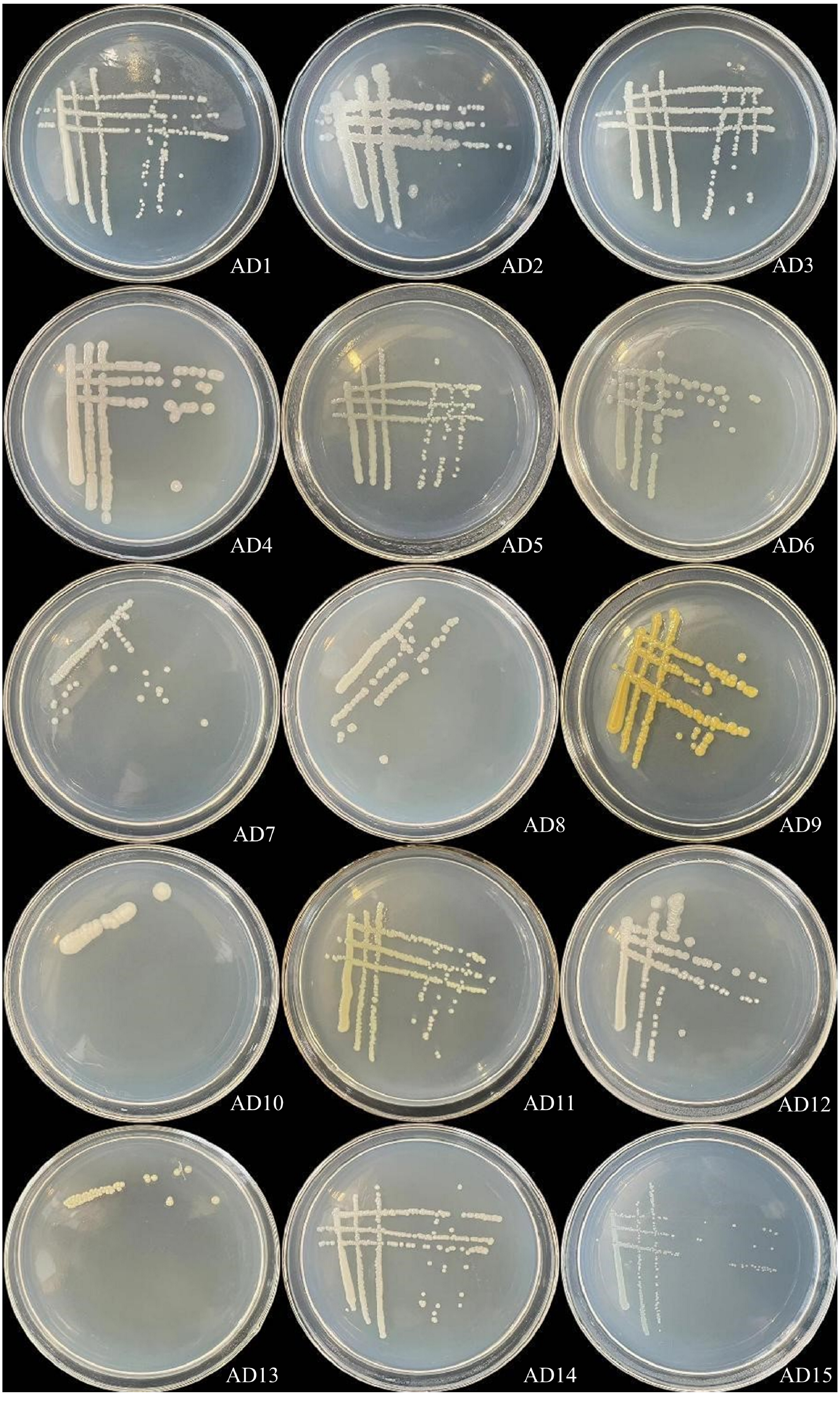

Figure S1: Colony morphology of 15 saline-alkali-tolerant strains.

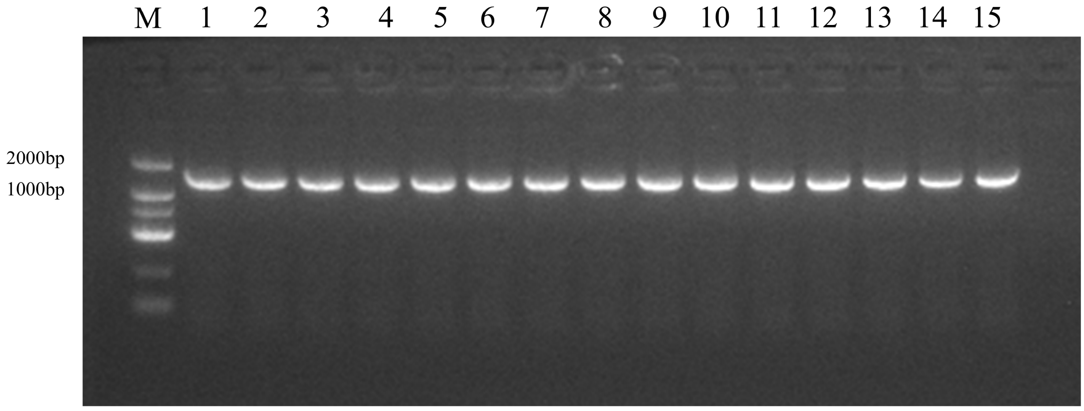

Figure S2: Agarose gel electrophoresis of PCR amplification products from 15 bacterial strains. Note: M: DNA molecular weight standard 2000; 1-15: Colony PCR products.

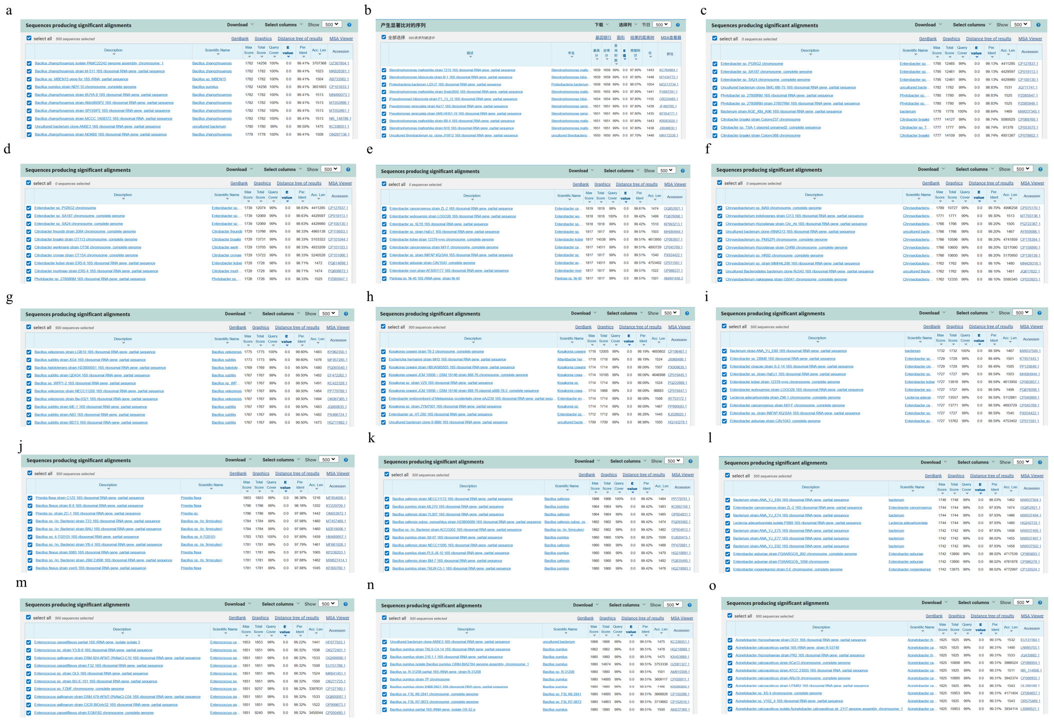

Figure S3: NCBI BLAST Analysis of 15 Isolates. Note: a-o are the comparison results of AD1-15.

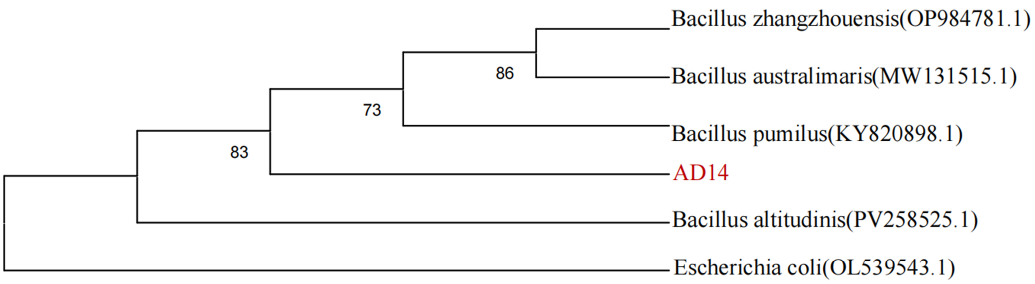

Figure S4: Phylogenetic tree of strain AD14 based on 16S rDNA sequence.
